# Production of extracellular fatty acid using engineered *Escherichia coli*

**DOI:** 10.1186/1475-2859-11-41

**Published:** 2012-04-03

**Authors:** Hui Liu, Chao Yu, Dexin Feng, Tao Cheng, Xin Meng, Wei Liu, Huibin Zou, Mo Xian

**Affiliations:** 1 Key Laboratory of Biofuel, Qingdao Institute of Bioenergy and Bioprocess Technology, Chinese Academy of Sciences, Qingdao, 266101, China

**Keywords:** Extracellular fatty acid, Extraction, Cultivation, Escherichia coli, Strain improvement

## Abstract

**Background:**

As an alternative for economic biodiesel production, the microbial production of extracellular fatty acid from renewable resources is receiving more concerns recently, since the separation of fatty acid from microorganism cells is normally involved in a series of energy-intensive steps. Many attempts have been made to construct fatty acid producing strains by targeting genes in the fatty acid biosynthetic pathway, while few studies focused on the cultivation process and the mass transfer kinetics.

**Results:**

In this study, both strain improvements and cultivation process strategies were applied to increase extracellular fatty acid production by engineered *Escherichia coli*. Our results showed overexpressing ‘TesA and the deletion of *fadL* in *E. coli* BL21 (DE3) improved extracellular fatty acid production, while deletion of *fadD* didn’t strengthen the extracellular fatty acid production for an undetermined mechanism. Moreover, the cultivation process controls contributed greatly to extracellular fatty acid production with respect to titer, cell growth and productivity by adjusting the temperature, adding ampicillin and employing on-line extraction. Under optimal conditions, the *E. coli* strain (pACY-*‘tesA*-Δ*fadL*) produced 4.8 g L^−1^ extracellular fatty acid, with the specific productivity of 0.02 g h^−1^ g^−1^dry cell mass, and the yield of 4.4% on glucose, while the ratios of cell-associated fatty acid versus extracellular fatty acid were kept below 0.5 after 15 h of cultivation. The fatty acids included C12:1, C12:0, C14:1, C14:0, C16:1, C16:0, C18:1, C18:0. The composition was dominated by C14 and C16 saturated and unsaturated fatty acids. Using the strain pACY-*‘tesA*, similar results appeared under the same culture conditions and the titer was also much higher than that ever reported previously, which suggested that the supposedly superior strain did not necessarily perform best for the efficient production of desired product. The strain pACY-*‘tesA* could also be chosen as the original strain for the next genetic manipulations.

**Conclusions:**

The general strategy of metabolic engineering for the extracellular fatty acid production should be the cyclic optimization between cultivation performance and strain improvements. On the basis of our cultivation process optimization, strain improvements should be further carried out for the effective and cost-effective production process.

## Background

Clean and renewable transportation fuels such as microbial biodiesel are greatly pushed forward in order to cope with global warming and energy shortage. Microbial fatty acid might become one of the potential feedstock for biodiesel production in the future since it has the following advantages: renewability, short production cycle, less labor requirement, less affection by venue, season and climate, and easier to scale up [1,2]. Prior to the conversion to biodiesel via esterification, microbial fatty acids have to be separated from cells through a series of energy-intensive steps such as cell harvest, drying and solvent extraction [3]. Among the energy-intensive processes, the cost of cell harvest usually accounts for 70–80% of total cost of biofuel production [4,5]. In order to skip these separation processes, the microbial production of extracellular fatty acid is receiving more concerns recently [3].

Although the mechanism of fatty acid secretion has not been illustrated definitely yet, it was reported that the fatty acid export out of cells could be through diffusion by concentration driven or through transportation by carrier proteins such as FadL, MsbA and so on [6-8]. Therefore, the process optimization could be employed to improve extracellular fatty acid production by increasing the driving force of fatty acid diffusion across the membrane. Furthermore, we could also enhance the extracellular fatty acid production in engineered *E. coli* by expressing the thioesterase or deleting the Fad gene family. There is now ample evidence that fatty acid export out of cells is relevant with the thioesterase gene and Fad gene family. The expression of full-length acyl-ACP thioesterase cDNA from *Umbellularia californica* in the *E. coli* fatty acid-degradation mutant strain (*fadD*^-^) resulted in the secretion of free fatty acid into medium [9]. The mutant of *Saccharomyces cerevisiae* deficient in acyl-CoA synthetases could secrete fatty acid out of cells with a maximum titer of 200 μmol L^−1^[10]. An *E. coli* fatty acid producing strain could produce 2.5 g L^−1^ of total fatty acid with <10% of the fatty acid pool secreted into the supernatant medium, which was constructed with four genotypic changes (deletion of the *fadD* gene, overexpression of endogenous ACC and ‘TesA as well as heterologous plant thioesterase) [11]. To avoid costly biomass recovery, the cyanobacteria was modified for fatty acid production by adding thioesterase genes and weakening polar cell wall layers and the extracellular fatty acid titer was 197 mg L^−1^[5].

Besides the traditional oleaginous microorganisms like microalgae, fungi and yeast, the industrial strain *E. coli* is becoming a new focus for fatty acid production and some breakthroughs have been made recently [12]. The formation of malonyl- CoA from acetyl- CoA catalyzed by acetyl- CoA carboxylase (ACC) is the first key rate-limiting step of fatty acid biosynthetic pathway [13,14]. The overexpression of ACC in *E. coli* has led to an increase in the rate of fatty acid synthesis [13]. Expression of bacterial or plant acyl-ACP thioesterases can reduce the cellular acyl-ACP concentration, decrease the feedback inhibition of fatty acyl-ACP and increase fatty acid production in *E. coli*[9,13,15,16]. A 7-fold increase of total fatty acid production was observed by eliminating β-oxidation in *E. coli*, overexpressing ACC and expressing plant acyl-ACP thioesterases from *U. californica* in low copy number plasmids, and the fatty acid was successfully converted to alkane by a catalytic reaction. In the *fadD* deleted strain, coexpression of three genes, thioesterases from *Cinnamomum camphorum* and *E. coli*, as well as ACC from *E. coli* resulted in 20-fold enhancement of total fatty acid production in shake flasks. 2.5 g L^−1^ fatty acids were produced by this engineered strain in fed-batch cultivation, but no cultivation optimization was further conducted [11]. Although many efforts have been done to increase fatty acid production by targeting the genes in the fatty acid biosynthetic pathway, the productivity (4.5 g L^−1^ d^−1^) is still not satisfied for the scale-up application [17].

Generally speaking, the construction of genetically engineered strains for fatty acid production only focused on the terminal pathway, such as the overexpression of the rate-limiting enzyme and removing feedback inhibitions in the fatty acid biosynthetic pathway. However, these approaches did not always result in great increment because the kinetic fermentative behavior of strains and the integrated function of the whole metabolic network were neglected [18-20]. The general strategy of metabolic engineering for the extracellular fatty acid production should be the cyclic optimization between cultivation performance and strain improvements [19]. Using developed strains, cultivation process should be performed. On the basis of cultivation performance, further metabolic engineering should be carried out for the strain improvement. The supposedly superior strain did not necessarily perform best for the efficient production of fatty acid [19]. Actually lots of factors could influence fatty acid production in the cultivation process such as cultivation temperature, which could affect the expression of genes, product transport out of cells, cell growth and productivity and so on [21-25]. Therefore, the bioprocess optimization for fatty acid production should be further considered.

In the present paper, to improve the production of extracellular fatty acid in metabolically engineered *E. coli*, a strain was first constructed by cytosolic overexpression of *E. coli* thioesterase. Then two derivative strains which contained the deletions of *fadD* and *fadL* respectively were constructed to inhibit the β-oxidation pathway or re-absorbance of extracellular fatty acid. Furthermore, considering the mass transfer kinetics during fatty acid diffusion out of cells, several cultivation strategies were employed to enhance extracellular fatty acid production, which included a two-stage control of cultivation temperature, an on-line integration of fatty acid separation with cultivation process, as well as ampicillin supplementation.

## Results and discussion

### Extracellular fatty acid production by *E. coli* pACY-‘*tesA* overexpressing native thioesterase

It was reported that the periplasmic expression of TesA (native *E. coli* thioesterase) in *E. coli* led to limited fatty acid accumulation and the cytosolic expression of ‘TesA (a ‘leadless’ version of TesA without the NH_2_-terminal 26 amino acid residues) could greatly contribute to the production and secretion of free fatty acids in cells [11,12,15,16,26]. Therefore, both *tesA* and *‘tesA* were cloned and transformed respectively into the strain BL21 (DE3) to promote the production of fatty acid. The overexpression of ‘TesA resulted in increment of both cell-associated fatty acid and extracellular fatty acid production in shake flasks (Table 1), which was similar to previous findings [12].

**Table 1:** **Extracellular fatty acid production by gene engineered*E. coli*under shake flask conditions**

**Strains**	**OD**_**600**_	**Extracellular fatty acid production (mg L**^**−1**^**)**	**Cell-associated fatty acid production (mg L**^**−1**^**)**
BL21 (DE3)	2.64 ± 0.32	5.5 ± 1.1	129.4 ± 3.0
BL21 (DE3) /pACYCDuet-1	2.81 ± 0.21	7.5 ± 2.1	117.6 ± 3.1
BL21 (DE3) /pACY-*tesA*	3.31 ± 0.26	25.2 ± 9.1	182.8 ± 6.9
BL21 (DE3) /pACY-*‘tesA*	3.48 ± 0.31	40 ± 5	539 ± 11

Values and error bars represent the mean and S.d of triplicate experiments.

**Figure 1:**
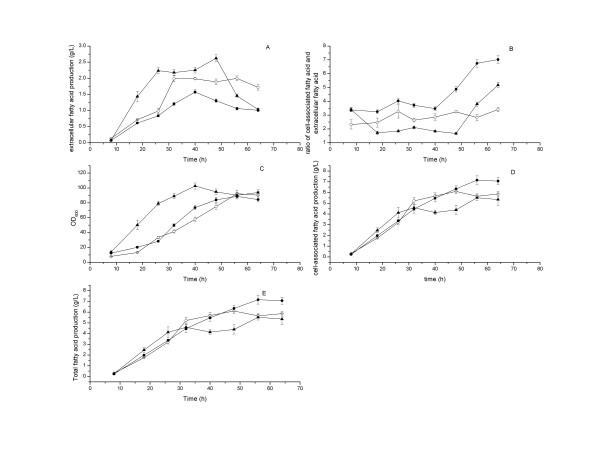
**Time courses of fatty acid production by*E. coli*pACY-*‘tesA*(*empty circle*), pACY-*‘tesA*-Δ*fadD*(*filled circle*) and pACY-*‘tesA*-Δ*fadL*(*filled upright triangle*) under standard cultivation conditions.** The cultivation temperature was maintained at 34°C from 8 h to 26 h, and then shifted to 30°C after 26 h. The pH was controlled at 7.0 ± 0.1 by automatic addition of 25% (w/w) ammonia water. The dissolved oxygen level was maintained at 20% ± 1% by adjusting agitation rate. The cultures were induced at OD_600_ = 8 by the addition of 0.5 mM IPTG. Values and error bars represent the mean and S.d of triplicate experiments.

Although the shake flasks are of outstanding importance for the practical application in screening projects, some disadvantages such as oxygen limitation, are also associated with shake bioreactors [27]. So the producer expressing ‘TesA was further investigated under fed-batch cultivation conditions in 5-L jar fermentors. As shown in Figure 1A, the extracellular fatty acid production reached 2.0 g L^-1^ with a specific productivity of 0.008 g h^−1^ g^−1^ dry cell and a yield of 1.9% (w/w) on glucose during the first 32 h of cultivation. The ratios of cell-associated fatty acid versus extracellular fatty acid varied between 2.3 and 3.4 from 8 to 64 h, which indicated that the cell-associated fatty acid concentrations were always much higher than the extracellular ones during the cultivation process.

Due to the extracellular fatty acid titers ever reported were much lower than 1 g L^−1^, the results above indicated that cytosolic *E. coli* thioesterase was one key enzyme for extracellular fatty acid production. Although ‘TesA was regarded as an essential enzyme for fatty acid production and secretion in *E. coli*, no one ever specified the performance of fatty acid producer expressing ‘TesA before, using ratio of cell-associated fatty acid versus extracellular fatty acid as a parameter.

### Extracellular fatty acid production using different strategies of cultivation temperature control

After 8 h of cultivation in a 5-L jar fermentor at 37°C, the *E. coli* strain pACY-*‘tesA* was induced with IPTG in 3 different control modes as follows: M1, 37°C for the whole process; M2, 30°C from 8 h to 72 h; standard cultivation conditions, 34°C from 8 h to 26 h, and then shifted to 30°C from 26 h to 72 h.

**Figure 2:**
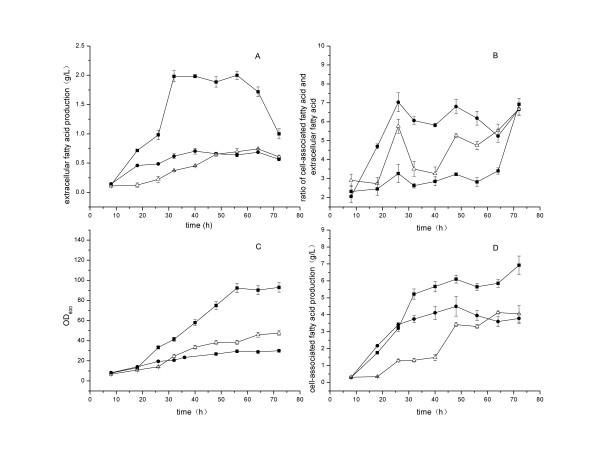
**Time courses of fatty acid production by*E. coli*pACY-*‘tesA*at different induction temperatures.** After 8 h of the initial cultivation at 37°C, the fatty acid producing strain pACY-*‘tesA* was induced with IPTG in 3 temperature control modes as follows: M1 (*filled circle*), 37°C for the whole process; M2 (*empty upright triangle*), 30°C from 8 h to 72 h; standard cultivation conditions (*filled square*), 34°C from 8 h to 26 h, and then shifted to 30°C from 26 h to 72 h. The pH was controlled at 7.0 ± 0.1 by automatic addition of 25% (w/w) ammonia water. The dissolved oxygen level was maintained at 20% ± 1% by adjusting agitation rate. Values and error bars represent the mean and S.d of triplicate experiments.

As shown in Figure 2, during the first 32 h of cultivation, the extracellular fatty acid production increased fast and the specific productivity reached 0.003 g h^−1^ g^−1^ dry cell (M1), 0.001 g h^−1^ g^−1^ dry cell (M2), 0.008 g h^−1^ g^−1^ dry cell (standard cultivation conditions). The yield reached 0.66% (M1), 0.41% (M2) and 1.9% (standard cultivation conditions). The maximal extracellular fatty acid production reached 0.6 g L^−1^ (M1), 0.4 g L^−1^ (M2) and 2.0 g L^−1^ (standard cultivation conditions) after 24 h of induction. The corresponding volumetric productivities were 0.5 g L^−1^ d^−1^ (M1), 0.2 g L^−1^ d^−1^ (M2) and 1.9 g L^−1^ d^−1^ (standard cultivation conditions), respectively. The two-stage mode (standard cultivation conditions) contributed greatly to fatty acid production with respect to fatty acid titer, cell growth and fatty acid productivity. We also showed the impact of cultivation temperature on the ratio of cell-associated fatty acid versus extracellular fatty acid in Figure 2B. Contrasted to M1 and M2, the extracellular fatty acid was greatly accumulated under standard cultivation conditions with a high titer of 2.0 g L^−1^ at 32 h and the ratios of cell-associated fatty acid versus extracellular fatty acid varied between 2.3 and 3.4 from 8 to 64 h.

Low induction temperatures 30°C or 25°C can increase the activity of recombinant enzymes at the expenses of the final yield and lower the inclusion bodies in genetically engineered *E. coli*[22,23,28]. Based on this consideration, previously fatty acid production by expressing thioesterase in *E. coli* has been conducted at low temperatures 30°C or 25°C [11,12,17,21]. However, a successful control of cultivation temperature has to balance the enzyme expression, cell growth and product formation, etc [22]. In this study, the higher induction temperatures (37°C, 34°C) in M1 and the two-stage mode could greatly contribute to the increments of extracellular fatty acid production and cell growth compared with 30°C in M2 in the first 26 h of cultivation (Figure 2). However, after 26 h the induction temperature 30°C in M2 and the two-stage mode increased the rates of extracellular fatty acids production and cell growth.

### Extracellular fatty acid production by *E. coli* pACY-‘*tesA* -Δ*fadD*

As shown in Figure 2A, the extracellular fatty acid production by *E. coli* pACY-*‘tesA* decreased after 56 h. Due to the extracellular fatty acid in the cultivation broth might be reabsorbed and consumed by the cells through β-oxidation pathway, we tried to address the problem with deletion of *fadD* in *E. coli* to stop the β-oxidation. The extracellular fatty acid production reached 1.2 g L^−1^, with the specific productivity of 0.003 g h^−1^ g^−1^ dry cell, the volumetric productivity of 1.1 g L^−1^ d^−1^ and the yield of 1.8% after 32 h of cultivation (Figure 1). In this case, the extracellular fatty acid produced by strain pACY-*‘tesA*-Δ*fadD* was lower than that of the original strain under cultivation conditions with respect to titer, specific productivity and yield. However, similar to other previous reports [9,11,12], we found the increase of total fatty acid production in *E. coli* with the deletion of *fadD*.

FadD (acyl-CoA synthetase) is the first enzyme in fatty acid β-oxidation pathway, and knockout of this enzyme could contribute to fatty acid accumulation. FadD can increase the concentration gradient between extracellular and intracellular fatty acid by converting fatty acid into acyl-CoA and contribute to the uptake of fatty acid [6]. In contrast, the deletion of *fadD* might increase the concentration of free fatty acid in the cytosol and enhance the export of fatty acid out of cells. However, our results suggested that the deletion of *fadD* could not improve the extracellular fatty acid production when the cell-associated fatty acid titer was high. Therefore, the extracellular fatty acid production was not only determined by the concentration gradient, but also related with other carrier proteins or some undetermined mechanisms such as the state of cells [9,10,26]. The reduced or flattened extracellular fatty acid production in Figures 1 and 2 could all be due to the state of cells, and the same trend was ever reported in *S. cerevisiae* previously [10].

Only in the *E. coli* fatty acid-degradation mutant strain K27 (*fadD*^-^), the expression of full-length acyl-ACP thioesterase cDNA from *U. californica* could result in the secretion of free fatty acid into medium [9]. The disparate results with respect to the effects of FadD on extracellular fatty acid production might be due to the different hosts and thioesterases.

### Extracellular fatty acid production by *E. coli* pACY-‘*tesA* -Δ*fadL*

We also tested the deletion of the outer membrane protein FadL of *E. coli*, and an obvious increase of extracellular fatty acid was observed under fed-batch cultivation conditions. After 32 h of cultivation, the extracellular fatty acid production reached 2.2 g L^−1^, with the specific productivity of 0.004 g h^−1^ g^−1^ dry cell, the volumetric productivity of 2.1 g L^−1^ d^−1^ and the yield of 2.6% (Figure 1).

The outer membrane protein FadL of *E. coli* is a fatty acid transporter membrane protein, which is responsible for the import of long chain fatty acid across the cytoplasmic membrane into cells [29]. Our results suggested that the deletion of *fadL* gene could greatly contribute to the extracellular fatty acid production.

### Extracellular fatty acid production using on-line integration of separation with cultivation

We also attempted to use on-line extraction during cultivation process as it was successfully applied in the production of organic acids and amino acids [30,31]. Considering the low water solubility, no tendency for emulsification, sufficient biocompatibility (Table 2), and high solubility of fatty acids (Table 3), tributyl phosphate (TBP) was chosen to be the solvent for the on-line extraction.

**Table 2:** **The inhibition of tributyl phosphate (TBP) on*E.coli*growth**

**TBP concentration (g L**^**-1**^**)**	**0**	**0.5**	**1**	**2**	**4**	**8**	**16**	**32**	**64**
OD_600_	2.11 ± 0.11	1.95 ± 0.23	1.89 ± 0.21	2.15 ± 0.24	2.05 ± 0.23	1.98 ± 0.12	1.76 ± 0.31	1.47 ± 0.15	1.15 ± 0.17

A single colony of *E. coli* pACY-‘*tesA* was inoculated to 3 mL M9 minimal medium supplemented with glucose (20 g L^−1^) as primary carbon source and 34 μg mL^−1^ chloramphenicol and cultured at 37°C for 12 h. One percent (v/v) inocula was added aseptically to 3 mL M9 minimal medium supplemented with glucose (20 g L^−1^) as primary carbon source, various concentrations of TBP and 34 μg mL^−1^ chloramphenicol, and was cultivated at 37°C and 180 rpm for 12 h. Values and error bars represent the mean and S.d of triplicate experiments.

**Table 3:** The solubility of fatty acid in tributyl phosphate (TBP)

**Fatty acid**	**C12:0**	**C14:0**	**C16:0**	**C18:0**
Solubility (g/L)	666.7 ± 0.1	444.4 ± 0.1	277.80 ± 0.06	100.00 ± 0.05

The solubility of fatty acid in TBP was determined by gravimetric method at 20°C. Values and error bars represent the mean and S.d of triplicate experiments.

**Figure 3:**
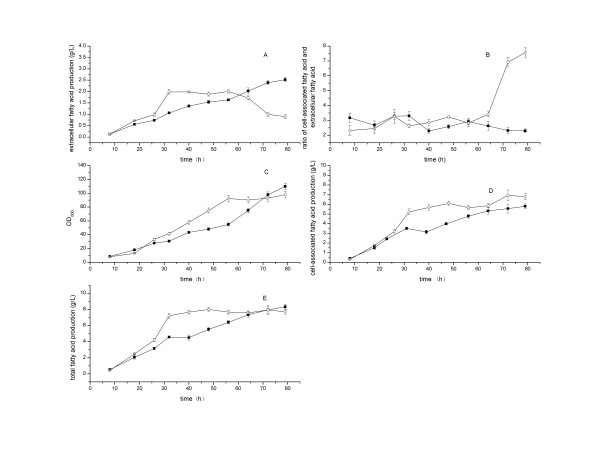
**Time courses of fatty acid production by*E. coli*pACY-*‘tesA*with integrated fatty acid separation in the cultivation process under standard cultivation conditions.** The cultivation temperature was maintained at 34°C from 8 h to 26 h, and then shifted to 30°C after 26 h. The pH was controlled at 7.0 ± 0.1 by automatic addition of 25% (w/w) ammonia water. The dissolved oxygen level was maintained at 20% ± 1% by adjusting agitation rate. The cultures were induced at OD_600_ = 8 by the addition of 0.5 mM IPTG. The on-line extraction unit was started and the flow rate of extraction cycle was maintained at 40 mL min^-1^ after 10 h of post-induction time. *Empty circle* and *filled square* indicate unintegrated and integrated fatty acid separation in the cultivation process, respectively. Values and error bars represent the mean and S.d of triplicate experiments.

The standard cultivation conditions and the on-line integration of separation with cultivation process were employed. As shown in Figure 3, the integrated extraction increased the extracellular fatty acid production to 2.5 g L^−1^ at 79 h, with the volumetric productivity of 0.85 g L^−1^ d^−1^, the specific productivity of 0.001 g h^−1^ g^−1^dry cell mass, and the yield of 0.96% on glucose. The extracellular fatty acid production kept increasing even after 40 h of cultivation, and the ratios of cell-associated fatty acid versus extracellular fatty acid were reduced to 2.3 at 79 h.

The on-line integration of separation with cultivation process was more effective than the unintegrated process for the extracellular fatty acid production. The decline in extracellular fatty acid production seemed inevitable after 56 h in the unintegrated cultivation processes (Figure 3). In contrast, the on-line extraction could increase the extracellular fatty acid production 2.8 times at 79 h. Although the contribution of on-line extraction to the total fatty acid was limited, the on-line integration of separation with cultivation process could still be a potential economical route for extracellular fatty acid production, because the *in situ* product removal (ISPR) could increase the product yield, reduce process time and cut down running costs and capital expenditure [32].

Lennen et al. used alkane as an extractant that was directly mixed with cultivation broth for extracellular fatty acid production in *E. coli*. The resulting emulsions were involved in a series of separation processes. Moreover, the experiments were performed at shake-flask level, and the extracellular fatty acid production was not clearly indicated [21]. In our case, the extractant TBP and the external cycle system were employed for the on-line extraction, which avoided the emulsification and the separation of emulsion.

The on-line extraction was necessary for the extracellular fatty acid production especially in the case of high fatty acid titer in the medium. Due to fatty acids accumulation in the cultivation broth, a lot of foam formed in the middle and late periods of cultivation and the huge fluctuation of dissolved oxygen concentration happened, which caused antifoam consumed too much and had negative effects on cultivation process control. The on-line extraction could increase the stability of cultivation process control through reducing the fatty acid titer in medium.

### Extracellular fatty acid production by ampicillin supplementation in medium

**Figure 4:**
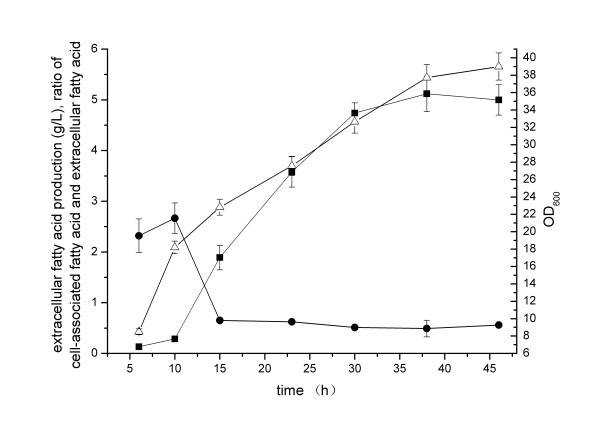
**Time courses of fatty acid production by*E. coli*pACY-*‘tesA*with addition of ampicillin in the cultivation process.** Integrated fatty acid separation and standard cultivation conditions were employed. The cultivation temperature was maintained at 34°C from 8 h to 26 h, and then shifted to 30°C after 26 h. The pH was controlled at 7.0 ± 0.1 by automatic addition of 25% (w/w) ammonia water. The dissolved oxygen level was maintained at 20% ± 1% by adjusting agitation rate. The cultures were induced at OD_600_ = 18 by the addition of 0.5 mM IPTG and 5 U mL^−1^ ampicillin. The on-line extraction unit was started and the flow rate of extraction cycle was maintained at 40 mL min^−1^ after 10 h of post-induction time. *Filled square*, *filled circle*, and *empty upright triangle* indicate extracellular fatty acid production, ratio of cell-associated fatty acid and extracellular fatty acid and OD_600_, respectively. Values and error bars represent the mean and S.d of triplicate experiments.

Ampicillin dose over 5 U mL^−1^ in shake flasks resulted in some inhibition on cell growth, while the ratios of cell-associated fatty acid versus extracellular fatty acid were reduced. So we investigated the effects of ampicillin supplementation with 5 U mL^−1^ as an example on the extracellular fatty acid production using *E. coli* pACY-*‘tesA* under fed-batch cultivation conditions. The standard cultivation conditions and the on-line integration of separation with cultivation process were employed in this case. As shown in Figure 4, the extracellular fatty acid production reached 5.1 g L^−1^ at 38 h with the volumetric productivity of 4.3 g L^−1^ d^−1^, the specific productivity of 0.02 g h^−1^ g^−1^dry cell mass, the yield of 4.1% on glucose. The ratios of cell-associated fatty acid versus extracellular fatty acid were kept below 0.7 after 15 h of cultivation. Obviously the addition of ampicillin in the cultivation broth enhanced the fatty acid secretion.

Cell envelopes were the barrier to fatty acid export out of cells. Ampicillin supplementation could positively affect the extracellular fatty acid production by alleviating this barrier in our case. A successful example was given previously in glutamate production by *Corynebacterium glutamicum*. In their case, ampicillin destroyed the integrity of cell walls by its addition in cultivation broth, enhanced the glutamate export out of cells and contributed to the overproduction of glutamate successfully [33].

Due to ampicillin cost and waste water treatment, the cost could be high for fatty acid production with the ampicillin addition in the cultivation process, which was not feasible for biofuel application. In order to reduce the cost and avoid using ampicillin, the deletion of the peptidoglycan assembly protein (PBP2) in *E. coli* pACY-*‘tesA* could be employed. Through binding the PBP2 in *E. coli*, ampicillin can inhibit the wall peptidoglycan assembly and weaken the polar cell wall layers [34,35]. So, the direct deletion of PBP2 in *E. coli* could also weaken the wall peptidoglycan layers and enhance the extracellular fatty acid production. This strategy has already been confirmed in genetically modified cyanobacteria [5].

Actually the chloramphenicol has to be added in the medium for plasmid stabilization in this study. However, antibiotics addition is definitely not a good option for producing low cost fatty acids for biofuel application. In order to avoid antibiotics addition in the production of biochemical products, various strategies such as addiction systems and chromosomal engineering have been implemented to construct engineering robust microbes. To avoid using the antibiotics for plasmid stabilization, addiction systems were developed by exploiting specific auxotrophic mutations and the vectors carrying the corresponding deleted genes [36,37]. The chemically inducible chromosomal evolution (CIChE) is a plasmid-free, high gene copy expression system for engineering *E. coli*, which only requires targeted genomic integration methods and a *recA* homolog [38]. Therefore, the antibiotics addition could be avoided for the low cost fatty acid production with these strategies mentioned above in the future.

### Extracellular fatty acid production by *E. coli* pACY-‘*tesA* -Δ*fadL* under optimal cultivation conditions

**Figure 5:**
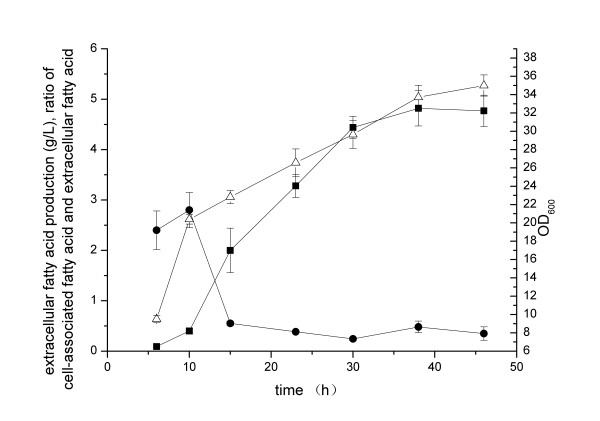
**Time courses of fatty acid production by*E. coli*pACY-*‘tesA*-Δ*fadL*with addition of ampicillin in the cultivation process.** Integrated fatty acid separation and standard cultivation conditions were employed. The cultivation temperature was maintained at 34°C from 8 h to 26 h, and then shifted to 30°C after 26 h. The pH was controlled at 7.0 ± 0.1 by automatic addition of 25% (w/w) ammonia water. The dissolved oxygen level was maintained at 20% ± 1% by adjusting agitation rate. The cultures were induced at OD_600_ = 18 by the addition of 0.5 mM IPTG and 5 U mL^−1^ ampicillin. The on-line extraction unit was started and the flow rate of extraction cycle was maintained at 40 mL min^−1^ after 10 h of post-induction time. *Filled square*, *filled circle*, and *empty upright triangle* indicate extracellular fatty acid production, ratio of cell-associated fatty acid and extracellular fatty acid and OD_600_, respectively. Values and error bars represent the mean and S.d of triplicate experiments.

Based on the above investigations, *E. coli* pACY-*‘tesA*-Δ*fadL* was finally employed to produce extracellular fatty acid with addition of 5 U mL^−1^ ampicillin in the cultivation process, while integrated fatty acid separation and standard cultivation conditions were employed. The time courses of fatty acid production were shown in Figure 5. The extracellular fatty acid production reached 4.8 g L^−1^ at 38 h with the volumetric productivity of 4.1 g L^−1^ d^−1^, the specific productivity of 0.02 g h^−1^ g^−1^ dry cell mass, the yield of 4.4% on glucose. The ratios of cell-associated fatty acid versus extracellular fatty acid were kept below 0.5 after 15 h of cultivation.

Using the strain pACY-*‘tesA*, similar results appeared under the same culture conditions and the titer was also much higher than that ever reported previously [5,9-11], which suggested that the supposedly superior strain did not necessarily perform best for the efficient production of desired product [19]. The strain pACY-*‘tesA* could also be chosen as the original strain for the next genetic manipulations.

In order to rule out the possibility of increasing extracellular fatty acid due to loss of cell viability, the free fatty acid fraction of extracellular fatty acid in the supernatant was determined and approximately 90% of extracellular fatty acid was in the free form. The results suggested the loss of cell viability did exist in the cultivation process, but the improvement of extracellular fatty acid was predominately due to the secretion of free fatty acid out of cells. The increasing trend of OD also indicated that the growth rate of *E. coli* pACY-*‘tesA*-Δ*fadL* was far beyond the inevitable loss of cell viability during the cultivation (Figure 5).

Fatty acids produced by *E. coli* pACY-*‘tesA*-Δ*fadL* were analyzed by GC-MS and listed in Table 4. The fatty acids included: C12:1, C12:0, C14:1, C14:0, C16:1, C16:0, C18:1, and C18:0. During 15 h to 38 h of cultivation, the proportions of the same length fatty acids remained relatively stable in the cell-associated fatty acid and the extracellular ones. And the composition was dominated by C14 and C16 saturated and unsaturated fatty acids.

**Table 4:** **Fatty acids produced by*E. coli*pACY-*‘tesA*-Δ*fadL*with addition of ampicillin in the cultivation process**

**fatty acids**	**Relative content of extracellular fatty acid (%)**				**Relative content of cell-associated fatty acid (%)**			
	15 h	23 h	30 h	38 h	15 h	23 h	30 h	38 h
C12:1	8.27	8.33	8.02	8.13	6.60	6.79	6.89	8.35
C12:0	8.00	6.16	6.51	7.51	2.46	4.20	5.37	5.24
C14:1	14.96	22.79	21.13	17.84	24.42	16.96	18.00	27.67
C14:0	9.28	10.67	11.11	12.47	7.80	6.91	8.46	9.67
C16:1	16.33	8.47	8.26	7.48	15.47	29.51	23.04	10.77
C16:0	25.21	31.69	33.18	35.35	28.06	19.89	24.80	28.30
C18:1	7.29	0.86	2.28	2.52	2.36	8.22	5.68	2.28
C18:0	10.68	11.03	9.50	7.72	12.83	7.51	7.76	7.72

Integrated fatty acid separation and standard cultivation conditions were employed.

These medium and long chain fatty acids could cause severe foaming problem during the cultivation process. The more fatty acids were produced, the more serious the foaming problem was. If the foam could not be controlled, the cultivation process would not continue normally either. In order to cope with the foaming problem, we increased the running frequency of anti-foam pump, lowered the upper surface of the foam detected by the anti-foam probe and raised the concentration of antifoam. Besides, the on-line extraction was also much helpful to solve the foaming problem through reducing the fatty acid titer in medium. Considering of the possible negative influence of reducing aeration rate on the cell metabolism for fatty acid production, the strategy of reducing aeration rate to control foam was not employed in the cultivation process.

## Conclusions

Several strategies of strain improvements and cultivation process control were successfully applied to increase extracellular fatty acid production, using ratio of cell-associated fatty acid versus extracellular fatty acid as a key parameter. The results showed ‘TesA overexpression, *fadL* deletion, the two-stage control of cultivation temperature, ampicillin supplementation and on-line extraction contributed greatly to extracellular fatty acid production. Thus, the general strategy of metabolic engineering for the extracellular fatty acid production should be the cyclic optimization between cultivation performance and strain improvements. On the basis of our cultivation process optimization, strain improvements should be further carried out for the effective and cost-effective production process.

## Methods

### Plasmid and strain construction

The native *E. coli* thioesterase (TesA) gene and ‘TesA (a ‘leadless’ version of TesA) gene were amplified by PCR from *E. coli* K-12 (ATCC10798) genomic DNA using primers *tesA*-L and *tesA*-R, *‘tesA*-L and *‘tesA*-R, respectively. The amplified TesA gene was inserted into pACYCDuet-1 (Novagen) between the *Nco*I and *BamH*Isites to generate pACY-*tesA*. The amplified ‘TesA gene was inserted into pACYCDuet-1 (Novagen) between the *EcoR*I and *Sal*I sites to generate pACY-*‘tesA*. *E. coli* BL21 (DE3) (Invitrogen) was used as a host for the expression of the TesA and ‘TesA genes. The primers used above were listed in Table 5. Bacterial strains and plasmids used in this study were listed in Table 6.

**Table 5:** Primers used in the study

**Primer name**	**Sequence (5′-3′)**
*tesA*-L	CATGCCATGGTTATGATGAAC TTCAACAATGTTTTCC
*tesA*-R	CGCGGATCCTTTATGAGTCA TGATTTACTAAAGGC
*‘tesA*-L	CGGAATTCGGCGGACACGTTA TTGATTCTGGG
*‘tesA*-R	ACGCGTCGACTTATGAGTCATGAT TTACTAAAGGC
*fadD*-IBS	AAAAAAGCTTATAATTATCCTTAGGATTCCTGCGCGTGCGCCCAGATAGGGTG
*fadD*-EBS1d	CAGATTGTACAAATGTGGTGATAACAGATAAGTCCTGCGCATTAACTTACCTTTCTTTGT
EBS2-fadD	TGAACGCAAGTTTCTAATTTCGATTAATCCTCGATAGAGGAAAGTGTCT
*fadL*-IBS	AAAAAAGCTTATAATTATCCTTAGGTTACAACCTGGTGCGCCCAGATAGGGTG
*fadL*-EBS1d	CAGATTGTACAAATGTGGTGATAACAGATAAGTCAACCTGACTAACTTACCTTTCTTTGT
*fadL*-EBS2	TGAACGCAAGTTTCTAATTTCGATTTAACCTCGATAGAGGAAAGTGTCT
EBS universal	CGAAATTAGAAACTTGCGTTCAGTAAAC

**Table 6:** Bacterial strains and plasmids used in this study

**Strain / plasmid**	**Relevant genotype / property**	**Source / reference**
Strains		
*E. coli* BL21(DE3)	F^-^*ompT hsdS*_*B*_(*r*_*B*_^*-*^*m*_*B*_^*-*^) *gal dcm rne131*(*DE3*)	Invitrogen
*E. coli* K-12	Type strain	ATCC
*E. coli* pACY-*tesA*	BL21(DE3) / pACY-*tesA*	This study
*E. coli* pACY-*‘tesA*	BL21(DE3) / pACY-*‘tesA*	This study
*E. coli* pACY-*‘tesA*-Δ*fadD*	BL21(DE3) Δ*fadD* / pACY-*‘tesA*	This study
*E. coli* pACY-*‘tesA*-Δ*fadL*	BL21(DE3) Δ*fadL* / pACY-*‘tesA*	This study
*E. coli* pACYCDuet-1	BL21 (DE3) /pACYCDuet-1	This study
Plasmids		
pACYCDuet-1	P15A origin, *lacI*^*q*^, T7 promoter, Cm^R^	Novagen
pACY-*tesA*	pACYCDuet-1 derivative carrying *tesA* gene, T7 promoter, Cm^R^	This study
pACY-*‘tesA*	pACYCDuet-1 derivative carrying ‘*tesA* gene (without leading sequence), T7 promoter, Cm^R^	This study

According to the protocol of the TargeTron Gene Knockout System Kit (Sigma-Aldrich), the retargeted intron DNA fragments *fadD*-intron and *fadL*-intron were amplified by overlap PCR using primer set 1 (*fadD*-IBS, *fadD*-EBS1d, *fadD*-EBS2 and EBS universal primer) and primer set 2 (*fadL*-IBS, *fadL*-EBS1d, *fadL*-EBS2 and EBS universal primer), respectively. These primers listed in Table 5 were designed using the TargeTron prediction program (http://www.Sigma-Aldrich.com/ Targetronaccess). The two resultant DNA fragments were cloned into the *Hind*III and *BsrG*I restriction sites of pACD4K-C for the deletion of *fadD* and *fadL* in *E. coli* BL21 (DE3) (Invitrogen). The resultant strains were used as hosts for the expression of the ‘TesA gene.

### Cultivation conditions in shake flasks

A single colony of *E. coli* pACY-‘*tesA* or pACY-*tesA* was inoculated to 5 mL M9 minimal medium (15.12 g L^−1^ Na_2_HPO_4_·12H_2_O, 3 g L^−1^ KH_2_PO_4_, 1 g L^−1^ NH_4_Cl, 0.5 g L^−1^ NaCl) supplemented with 34 μg mL^−1^ chloramphenicol and cultured overnight at 37°C [39]. Three percent (v/v) inocula was added aseptically to a 500-mL shake flask containing 50 mL M9 minimal medium supplemented with glucose (20 g L^-1^) as primary carbon source and 34 μg mL^−1^ chloramphenicol, and was cultivated at 37°C and 180 rpm. The cultures were induced at an optical density (OD_600_) of 0.6 by the addition of 0.5 mM isopropylthiogalactoside (IPTG) at 30°C and samples were collected 20 h post-induction for fatty acids analysis. To evaluate the effects of ampicillin on the biomass and fatty acids secretion, various concentrations of ampicillin were added in culture broth at OD_600_ = 1.4.

### Fed-batch cultivation

A single colony of *E. coli* pACY-*‘tesA*, pACY-*‘tesA*-Δ*fadD* or pACY-*‘tesA*-Δ*fadL* was inoculated to a 500-mL shake flask containing 25 mL M9 minimal medium supplemented with glucose (20 g L^-1^) as primary carbon source and 34 μg mL^−1^ chloramphenicol, and was cultivated at 37°C, 180 rpm for 12 h. Three percent (v/v) inocula was added aseptically to 5-L jar fermentors (BIOSTAT Bplus 5 L, Sartorius stedim) containing 3 L medium consisted of the following components: 3 g (NH_4_)_2_SO_4_, 9 g K_2_HPO_4_·3H_2_O, 9.45 g MgSO_4_·7H_2_O, 5.7 g KCl, 3 g sodium citrate, 3 g citric acid, 60 g glucose, 15 mL corn steep liquid (corresponding to 0.47 g of nitrogen) and 3 mL trace elements (0.37 g (NH_4_)_6_Mo_7_O_24_·4H_2_O, 0.29 g ZnSO_4_·7H_2_O, 2.47 g H_3_BO_3_, 0.25 g CuSO_4_·5H_2_O, 1.58 g MnCl_2_·4H_2_O). The cultivation temperature was maintained at 34°C from 8 h to 26 h, and then shifted to 30°C from 26 h to 72 h unless specified according to different purposes. The pH was controlled at 7.0 ± 0.1 by automatic addition of 25% (w/w) ammonia water. The aeration rate was 3 vvm and dissolved oxygen level was maintained at 20% ± 1% by adjusting agitation rate. When the initial glucose was depleted, 800 g L^−1^ concentrated glucose was intermittently fed into the fermentor at a constant flow rate of 4.2 g L^−1^ h^−1^. The cultures were induced at OD_600_ = 8 by the addition of 0.5 mM IPTG and samples were collected at certain intervals for fatty acids analysis.

### Integration of fatty acids separation with fed-batch cultivation

**Figure 6:**
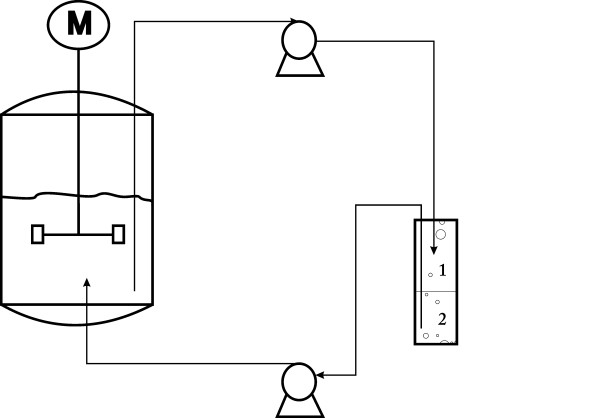
Principle of integrated fatty acids extraction with fed-batch cultivation. 1 and 2 indicate the TBP layer and the cultivation broth layer, respectively.

The 5-L jar fermentor (BIOSTAT Bplus 5 L, Sartorius stedim) was combined with an extraction unit using a 500-mL glass jar containing 200 mL tributyl phosphate (TBP) (Figure 6). The integrated fatty acids separation system in the fed-batch cultivation employed a flow cycle between the 5-L jar fermentor and the 500-mL extraction jar. The cultivation broth was pumped from the fermentor to the glass jar and pumped back into the fermentor after the extraction operation. The culture conditions were the same as the fed-batch cultivation. The on-line extraction unit was started and the flow rate of extraction cycle was maintained at 40 mL min^−1^ after 10 h of post-induction time. To evaluate the effects of ampicillin supplementation on the fatty acid production and secretion, the cultures were induced at OD_600_ = 18 by the addition of 0.5 mM IPTG and 5 U mL^−1^ ampicillin.

### Fatty acid extraction, saponification and methylation

5-mL samples of cell culture (three replicates for each sample) were centrifuged at 8000 rpm for 10 min to separate the cell-associated fatty acid and extracellular fatty acid. The extracellular fatty acid in the supernatants was extracted three times with 20 mL of chloroform/ methanol mixture (2:1, v/v). The cell pellets were resuspended in 1 mL distilled water and mixed with 20 mL chloroform/ methanol mixture (2:1, v/v) to obtain cell-associated fatty acid as previously described [9]. 100 μL of 10 g L^−1^ arachidic acid (Alfa Aesar) was added as the internal standard respectively. The chloroform layer was evaporated under a nitrogen stream and the dried fatty acid sample was used for the analysis of extracellular, cell-associated or free fatty acid.

For the extracellular or cell-associated fatty acid analysis, the dried fatty acid was redissolved in 2 mL of 20 g L^−1^ (w/v) NaOH in methanol/ water mixture (4:1, v/v) and the saponification reaction was incubated at 60°C for one hour. To methylate the fatty acids, 3 mL of BF_3_/ CH_3_OH (1:4, v/v) and 170 μL of 6 mol L^−1^ HCl were added and the reaction was incubated at 60°C for 30 min. The fatty acid methyl esters (FAMEs) were extracted twice using 1.5 mL of n-hexane and collected for analysis.

For the free fatty acid analysis, the dried fatty acid was redissolved in 500 μL of chloroform/ methanol mixture (2:1, v/v) and loaded on the thin-layer chromatography (TLC) plate. The TLC plate was developed with hexane-diethyl-ether-formic acid (70:30:0.2, v/v/v) [40]. The free fatty acid was identified and eluted out with chloroform and dried. To methylate the fatty acids, 3 mL of BF_3_/ CH_3_OH (1:4, v/v) and 170 μL of 6 mol L^−1^ HCl were added and the reaction was incubated at 60°C for 30 min. The fatty acid methyl esters (FAMEs) were extracted twice using 1.5 mL of n-hexane and collected for analysis.

### Gas chromatography/ mass spectrometry and gas chromatography of fatty acid methyl esters

Gas chromatography/ mass spectrometry (GC/MS) analysis was performed in a Thermo Fisher Trace GC Ultra-ITQ1100 system with a 30-m HP-5 ms column. The column temperature program was 100°C for 2 min, a ramp to 280°C at a rate of 10°C min^−1^ and 280°C for 3 min.

The gas chromatography (GC) analysis of FAMEs was performed in a Varian GC-450 system equipped with a 30-m HP-5 column and using nitrogen as carrier gas with a linear velocity of 1 mL min^−1^. GC elution conditions were as follows: 100°C for 2 min, a ramp to 250°C at a rate of 10°C min^−1^ and a final hold at 250°C for 5 min. The split ratio was 1:10 or 1:50. The quantification of fatty acids was achieved with reference to the internal standard (arachidic acid) and the response factors and averaged for all replicates.

### Calculations

The following equation was used to calculate the specific productivity [41].

(1) $$Q_{p}=\frac{p_{1}-p_{0}}{t_{1}-t_{0}}\times \frac{2}{x_{1}+x_{0}}$$

Where Q_p_, Specific production rate (g h^−1^ g^−1^ dry cell); p, Fatty acid concentration (g/L); t, Cultivation time (h); x, biomass (g/L).

Cell culture optical density was measured at 600 nm using a spectrophotometer ( Cary 50 UV–vis, VARIAN) and the dry cell weight was calculated according to the coefficient (one OD_600_ unit corresponded to 0.43 g L^−1^ of dry cell weight).

## Competing interests

The authors declare that they have no competing interests.

## Authors' contributions

HL and CY conceived of the study, participated in its design, carried out the process control studies and drafted the manuscript. DF participated in the coordination of this study, contributed to the GC-MS data analysis and helped to draft the manuscript. TC carried out the molecular genetic studies and the process control studies. XM carried out the molecular genetic studies. WL and HZ helped to draft the manuscript. MX conceived of the study, and participated in its design and coordination and helped to draft the manuscript. All authors read and approved the final manuscript.
